# Validation of administrative health data for the pediatric population: a scoping review

**DOI:** 10.1186/1472-6963-14-236

**Published:** 2014-05-22

**Authors:** Natalie J Shiff, Sadia Jama, Catherine Boden, Lisa M Lix

**Affiliations:** 1 Department of Pediatrics, College of Medicine, University of Saskatchewan, 103 Hospital Drive, SK S7N 0W8, Saskatoon, Canada; 2 Department of Community Health and Epidemiology, College of Medicine, University of Saskatchewan, 103 Hospital Drive, SK S7N 0W8, Saskatoon, Canada; 3 University Library, University of Saskatchewan, Room 1441, Leslie and Irene Dube Health Sciences Library, 104 Clinic Place, SK S7N 5E5, Saskatoon, Canada; 4 Department of Community Health Sciences, S113-750 Bannatyne Avenue, University of Manitoba, MB R3E 0W3, Winnipeg, Canada

## Abstract

**Background:**

The purpose of this research was to perform a scoping review of published literature on the validity of administrative health data for ascertaining health conditions in the pediatric population (≤20 years).

**Methods:**

A comprehensive search of OVID Medline (1946 - present), CINAHL (1937 - present) and EMBASE (1947 - present) was conducted. Characteristics of validation studies that were abstracted included the study population, health condition, topic of the validation (e.g., single diagnosis code versus case-finding algorithm), administrative and validation data sources. Inter-rater agreement was measured using Cohen’s *κ*. Extracted data were analyzed using descriptive statistics.

**Results:**

A total of 37 articles met the study inclusion criteria. Cohen’s *κ* for study inclusion/exclusion and data abstraction was 0.88 and 0.97, respectively. Most studies validated administrative data from the USA (43.2%) and Canada (24.3%), and focused on inpatient records (67.6%). Case-finding algorithms (56.7%) were more frequently validated than diagnoses codes alone (37.8%). Five conditions were validated in more than one study: diabetes mellitus, inflammatory bowel disease, asthma, rotavirus infection, and tuberculosis.

**Conclusions:**

This scoping review identified a number of gaps in the validation of administrative health data for pediatric populations, including limited investigation of outpatient populations and older pediatric age groups.

## Background

Administrative health data, which are generated through the routine delivery of health care programs [1], are rich sources of population-based information for research about population health and health services. However, these data were not originally intended for research, leading to many questions about their validity for this purpose. In particular, the use of diagnostic codes in these data, which are typically recorded using the World Health Organization’s International Classification of Diseases (ICD), to accurately identify patient populations with acute or chronic diseases has been the focus of multiple validation studies. These studies compare individual diagnostic codes or more complex case-finding algorithms based on combinations of diagnosis codes and other criteria in administrative health data to an external data source, such as survey data, medical charts, or laboratory test results [2,3]. Validation studies and systematic reviews of validation studies [4-6] have primarily focused on adult populations; there have been few validation studies conducted in pediatric populations. A recent review of the quality of validation studies underscored the importance of population-specific studies, because validity may be heterogeneous across populations [7].

Age may be particularly important in the assessment of diagnostic validity because pediatric and adult diseases often differ [7]. For example, only five percent of pediatric patients with juvenile idiopathic arthritis (formerly called juvenile rheumatoid arthritis) have a disease pattern similar to the pattern observed in adult patients with rheumatoid arthritis [8], which may result in discrepant diagnostic validity estimates between the two populations.

The purpose of this study was to synthesize the published literature on the validity of diagnoses recorded in administrative health data for the pediatric population (≤20 years). This study was conducted to identify gaps in the literature and opportunities for future research.

## Methods

### Type of study

Given that we did not identify any previous syntheses of diagnostic validation studies for administrative health data in the pediatric population, we conducted a scoping review, which is intended to: (a) map an area of study, (b) identify whether a full systematic review of the literature is warranted, (c) summarize and disseminate research, and (d) identify gaps in the literature [9,10]. The primary difference between a systematic review and a scoping review is that in the latter, study quality is not the focus of the evaluation [9,10], but in the former it is.

### Literature search

The literature searches were conducted on October 22, 2012. The following electronic databases were comprehensively searched: OVID Medline (1946 - present), CINAHL (1937 - present) and EMBASE (1947 - present). These databases have been used in other systematic reviews of validation studies about diagnostic codes in administrative health databases [7]. Medline is a major bibliographic database for clinical medicine and has its origins in North America. CINAHL primarily indexes the nursing and allied health journals, and includes mainly North American journals as well as some European, Asian, and Australasian journals. EMBASE is a major biomedical and pharmaceutical database that indexes international journals not represented in Medline or CINAHL.

Three conceptual groupings of terms were used to define the scope of this review: (a) validation study, (b) pediatric population and (c) administrative health data. A validation study can be characterized by its research method and outcome measures of sensitivity, specificity, predictive value and receiver operating characteristics. Administrative data include admissions records, discharge data/records/claims/abstracts, hospital records, outpatient records, inpatient records, physician claims, billing data and medical record linkage. Pediatric populations can be identified by age group (e.g., infant, child, adolescent) and pediatrics specialty.

A preliminary search of the published literature was conducted and the words in the title, abstract, and subject heading were used to develop the final search strategy. This strategy was developed for Medline first (Table 1), and then adapted for EMBASE and CINAHL. Key words and subject headings were combined using Boolean operators. No limits were placed on publication date or type (e.g., journal article, systematic review). The reference lists of all included articles were examined to identify additional articles that may have been missed during the database search.

**Table 1:** Medline search strategy for scoping review

**‘Validation study’ search terms**	**‘Pediatric population’ search terms**	**‘Administrative data’ search terms**
1. validation studies [MeSH]	7. exp pediatrics [MeSH]	10. medical record linkage [MeSH]
2. case definition*.mp.	8. *adolescent [MeSH] or exp child [MeSH] or exp infant [MeSH]	11. “discharge claim*”.ab, ti.
		12. discharge data.ab, ti.
3. case validation.mp.	9. 7 or 8	13. “administrative data*”.ab, ti.
4. “sensitivity and specificity” [MeSH] or “predictive value of tests” [MeSH] or roc curve [MeSH]		14. “hospital record*”.ab, ti.
5. valid*.ab, ti.		15. “outpatient record*”.ab, ti.
6. or/1-5		16. “inpatient record*”.ab, ti.
		17. “physician claim*”.ab, ti.
		18. “Clinical Coding” [MeSH]
		19. “International Classification of Diseases” [MeSH] with/sn [Statistics & Numerical Data] subheading
		20. (ICD9 or “ICD 9” or ICD-9).ab, ti.
		21. (ICD10 or “ICD 10” or ICD-10).ab, ti.
		22. “administrative billing code*”.ab, ti.
		23. hospital-discharge data.ab, ti.
		24. hospital billing data.ab, ti.
		25. “discharge code*”.ab, ti.
		26. “admissions record*”.ab, ti.
		27. “discharge record*”.ab, ti.
		28. “discharge abstract*”.ab, ti.
		29. or/10-28

Note: *MeSH* = medical subject heading; .mp = keywords search of title, abstract, heading word, table of contents, key concepts, original title, tests & measures; .ti, ab = keyword search of title, abstract only; quotations = a phrase search; exp = used with a MeSH term to include all narrower MeSH terms; *after keyword indicates truncation (e.g., code* will retrieve “codes”, “code”, “coded”); *with a MeSH term indicates that the term is a major topic of the article.

The bibliographic information (e.g., title, authors, abstract, subject headings, and website address [where applicable]) was imported into Refworks bibliographic management software for storage and removal of duplicate citations (http://www.refworks.com/).

### Selection and data extraction

Following the removal of duplicate citations, a training phase was used to ensure that study inclusion criteria were consistently applied for a randomly selected subset of approximately 5% of the studies. A citation was included if: (a) analyses were conducted for patients aged 0 to 20 years of age, (b) results of primary research were reported in peer reviewed publications, (c) it was published in English as translation resources were not available, and (d) it was a validation study of administrative health data. Administrative health data differ from registries in that the latter refer to data systems in which information about all cases of a specified disease in a given population are recorded [11]. Examples include cancer registries, birth defect registries, and twin registries. Studies about the validity of registries were not included in the scoping review.

Following the training phase, two authors (NS and SJ) applied the study inclusion criteria to another randomly selected sample of 23 studies, and kappa was calculated for the decision to include or exclude (yes or no). Both authors extracted data from this validation set using a standardized form. All data extracted by each of the respective authors were then coded and pooled, and kappa was calculated for the pooled results of the data extraction. Subsequently, one investigator (SJ) applied the inclusion criteria to all remaining studies and extracted data from the retained studies.

The abstracted information included characteristics of the citation (e.g., publication year), study population (e.g., country of origin, age group and gender), health condition(s) that were investigated, administrative health data (e.g., the diagnosis codes or case finding algorithms that were validated, type of data source, type of diagnostic coding system), and the external data used to conduct the validation.

### Statistical analyses

Inter-rater agreement was assessed using Cohen’s *κ*[12] for: (a) study inclusion and (b) data extraction. As well, 95% confidence intervals (CIs) were calculated. The data were analyzed using descriptive statistics, including frequencies and percentages.

## Results

**Figure 1:**
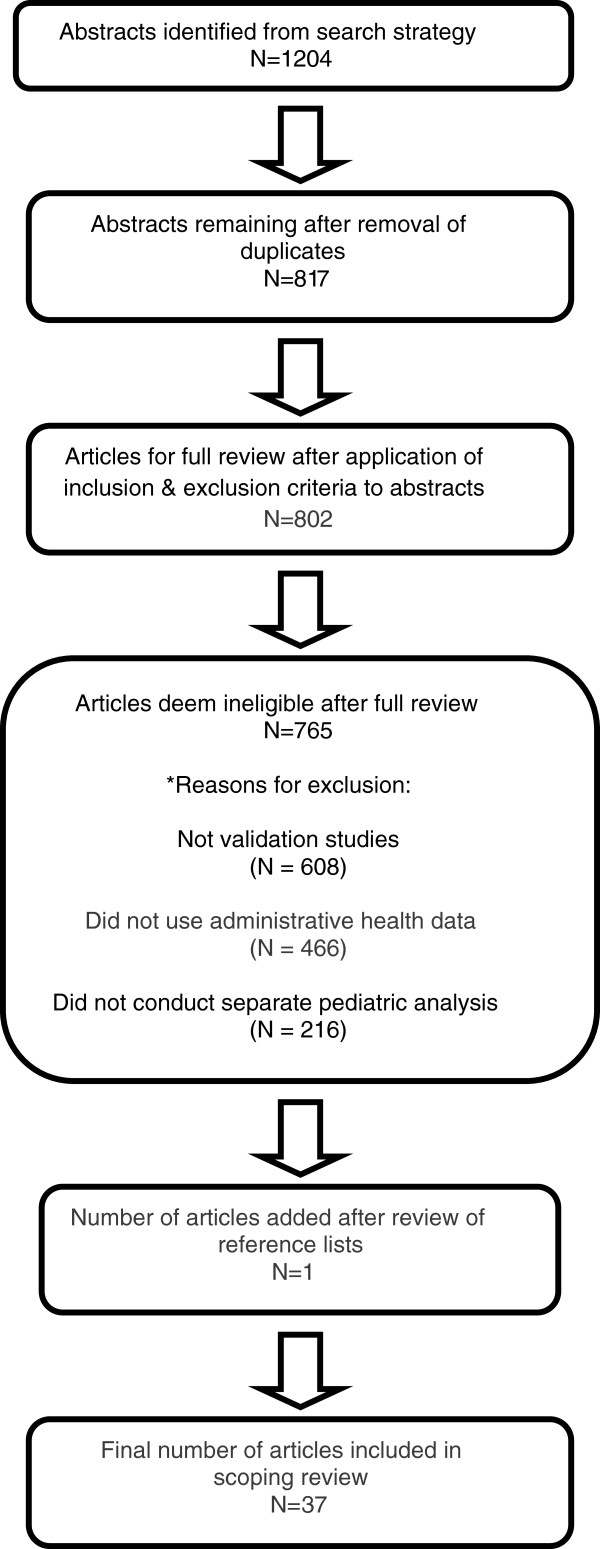
**Scoping review process.** The final review included 37 articles. *reasons for exclusion are not mutually exclusive.

A total of 1204 abstracts were identified by the literature search (Figure 1). After removing duplicates, 817 unique abstracts were screened for study inclusion. Fifteen were excluded based only on the title and abstract (1.8%). Thus, a total of 802 articles (98.2%) underwent full text review. Of this number, 765 (95.4%) were excluded for the following reasons (reasons are not mutually exclusive): 608 (75.8%) were not validation studies, 466 (58.1%) did not use administrative health data, and 216 (26.9%) did not conduct separate validation analyses for pediatric patients. Thirty-six articles met criteria for further analysis. A hand search of the reference lists of included studies identified one additional article, yielding a final sample of 37 articles. Cohen’s *κ* for study inclusion/exclusion and data abstraction was 0.88 (95% CI 0.72, 1.00) and 0.97 (95% CI 0.94, 0.99), respectively.

The characteristics of included studies are summarized in Table 2. Increasing numbers of validation studies were published over time, with 11 (29.7%) published between 2006 and 2010 and a further 12 (32.4%) identified between 2011 and 2012 (up to the end of the search period). Just over 40% of the studies (16 studies, 43.2%) were conducted using administrative health data from the United States, followed by Canada (9 studies, 24.3%). All validation studies included both males and females. There was a trend of smaller numbers of validation studies as age increased, with fewer studies (17 studies, 45.9%) including individuals aged 16 to 20 years.

**Table 2:** Characteristics of validation studies in the scoping review

**Study characteristic ( *N * = 37)**	***n *** **(%)**
***Year of publication***	
Prior to 2001	4 (10.8)
2001-2005	10 (27.0)
2006-2010	11 (29.7)
2011-2012	12 (32.4)
***Country of origin***	
USA	16 (43.2)
Canada	9 (24.3)
Denmark	3 (8.1)
New Zealand	3 (8.1)
China	2 (5.4)
United Kingdom	2 (5.4)
Other	2 (5.4)
***Age group *** **( *years *)**^**a**^	
0 - 5	29 (78.3)
6 - 10	21 (56.7)
11 - 15	29 (54.0)
16 - 20	17 (45.9)
***Condition validated***	
Diabetes	4 (10.8)
Inflammatory bowel disease	2 (5.4)
Asthma	2 (5.4)
Rotavirus infection	2 (5.4)
Tuberculosis	2 (5.4)
Other	25 (67.6)
***Topic of validation***	
Diagnosis codes only	14 (37.8)
Case-finding algorithm	21 (56.8)
Both	2 (5.4)
***Administrative data source***	
Inpatient	25 (65.8)
Outpatient	9 (23.7)
Emergency department	2 (5.3)
Pharmacy	2 (5.3)
***Diagnosis coding system***^**a**^	
ICD-9 or ICD-9-CM	28 (75.7)
ICD-10 or ICD-10-CA	8 (21.6)
Other	2 (5.4)
***Validation data source***^**a**^	
Medical chart	23 (62.2)
Disease registry	6 (16.2)
Clinical database	4 (10.8)
Laboratory data	4 (10.8)
Survey	2 (5.4)
***Validation measure***^**a**^	
Sensitivity	24 (64.8)
Specificity	20 (54.0)
PPV^b^	17 (45.9)
NPV^c^	8 (21.6)
Other^d^	4 (10.8)

^a^Categories are not mutually exclusive; ^b^*PPV* = positive predictive value; ^c^*NPV* = negative predictive value; ^d^Other = correlation coefficient, relative risk of association, kappa, percentage of agreement.

Slightly more than one-third of studies (14 studies, 37.8%) validated diagnosis codes, while more than half evaluated case-finding algorithms (21 studies, 56.8%), which use a combination of diagnosis codes and other criteria (e.g., procedure codes) to identify cases with the condition of interest. Two studies (5.4%) validated both diagnosis codes and case-finding algorithms.

Only five conditions were investigated in more than one study: diabetes (10.6%) [13-16], inflammatory bowel disease (5.4%) [17,18], asthma (5.4%) [19,20], rotavirus infection (5.4%) [21,22], and tuberculosis (5.4%) [23,24]. However, a diverse range of conditions were investigated in single studies, including obesity [25], vaccine-related illness [26], injuries [27], autism [28], febrile neutropenia in oncology patients [29], high risk conditions [30], dermatologic conditions [31-33], congenital anomalies [34], cardiac defects [35], respiratory illnesses excluding asthma [36-38], neurologic conditions [39], other gastrointestinal conditions [40-43], genitourinary conditions [44,45], serum sickness [46], thrombosis [47], maternal/perinatal conditions [48], and drug-related anaphylaxis [49].

Administrative health data sources that were validated consisted of inpatient (25 studies, 67.6%), outpatient (9 studies, 24.3%), and emergency room records (2 studies, 5.4%), as well as pharmacy data (2 studies, 5.4%). Studies that validated individual diagnoses or case-finding algorithms in a single database were most frequent (25 studies, 67.6%). Eighty percent (20 studies) of the 25 studies that validated data from a single administrative database used inpatient administrative data (54.1% of 37 studies), followed by outpatient administrative data (4 studies, 10.8% of 37 studies) and emergency department data (1 study, 2.7% of 37 studies). Twelve studies (32.4% of 37 studies) validated information from multiple, linked administrative data sources; five of these studies included inpatient records (13.5% of 37 studies) and five included outpatient records as one of the databases.

The main diagnosis coding systems that were validated included ICD-9 or ICD-9 CM (28 studies, 75.7%) and ICD-10 or ICD-10-CA (8 studies, 21.6%). The most frequent external data sources used to validate administrative data were medical charts (23 studies, 62.2%) and disease-specific registry data (6 studies, 16.2%). Other validation sources used included clinical databases, laboratory records, and survey data. Validation measures reported include sensitivity (24 studies, 64.8%), specificity (20 studies, 54.0%), positive predictive value (17 studies, 45.9%), and negative predictive value (8 studies, 21.6%).

## Discussion

The prevalence of chronic pediatric conditions has increased over recent decades; it is estimated that between 16% and 51% of children have at least one chronic condition [50-52]. Medical advances have improved survival for conditions that were once fatal, resulting in an increasing number of children with special healthcare needs [50-52]. In order to allow for resource planning and optimization of care, the long-term outcomes of children and youth with chronic conditions need to be determined, as does their healthcare utilization [52]. Administrative health data are an appropriate source to conduct long-term follow-up studies, but validation studies are important to ensure that true cases of disease can be ascertained in these data.

To the best of our knowledge, this is the first scoping review to describe validation studies of administrative health data in the pediatric population. Only a small number of pediatric validation studies were identified, whereas a recent systematic review about the quality of reporting of administrative data validation studies that included all age groups, identified 271 studies published prior to June 2009 [7], most of which focused on the adult population. The increasing number of pediatric validation studies in recent years suggests that there is growing recognition that pediatric populations are important to consider separately from adult populations when validating administrative health data. The vast majority of studies were conducted in North America, reflecting a general trend for validation studies [4-6]. Most of the 37 studies included in this scoping review validated complex case-finding algorithms that use a variety of information found in administrative health data to ascertain disease cases.

Case-finding algorithms typically take advantage of linked administrative health databases, whereas validation of individual diagnoses may only take place in a single administrative data source. In many administrative data systems, data linkage creates the opportunity to evaluate case-finding algorithms that will have sensitivity or specificity that is greater than what can be observed by examining a diagnosis in an unlinked database.

It is surprising that no validation studies were identified for common chronic pediatric conditions such as attention deficit/hyperactivity disorder and obesity [50]. Only diabetes, inflammatory bowel disease, asthma, tuberculosis, and rotavirus infection were validated in more than one setting. It is well known that diabetes can be ascertained from administrative health data with high specificity and sensitivity for adult populations, which may have contributed to increased interest in performing validation studies for this diagnosis in the pediatric population. In addition, with the growth in rates of juvenile diabetes, this is an important condition for chronic disease research and surveillance [50]. There is a gap in the literature for conditions validated in the adults but not in the pediatric setting. Rheumatoid arthritis is one example of a chronic condition for which several validation studies have been published in the adult population [53-55] but similar validation studies are lacking in the pediatric age group. In fact, no validation studies were found for chronic inflammatory arthritis in the pediatric population at the time of this scoping review.

While this scoping review has several strengths, including the breadth of citation databases investigated, the multiple health conditions that were included, and the range of characteristics of the studies that were examined, it does have some limitations. Only English language publications were included. Conference proceedings and articles that were not published in peer-review journals were excluded. Publication bias may affect the generalizability of the scoping review results. Nevertheless, these factors taken together are not likely to result in a large number of missing research studies, and hence cannot account for the relative dearth of pediatric administrative data validation studies that were identified.

For conditions with several published validation studies in the pediatric population, such as diabetes, disease specific systematic reviews evaluating the quality of studies should be examined, but only once more studies have been published; at present, there are too few validation studies in pediatric publications to warrant systematic reviews. Many pediatric conditions are treated primarily in an outpatient setting, and almost all chronic diseases in this population require at least some outpatient care, yet validation studies in this setting are lacking. Patients with milder disease or better access to outpatient-based services may never need hospitalization, and validation studies primarily based on inpatient data likely do not capture the true spectrum of chronic disease severity. Validation studies in the outpatient setting can be challenging to conduct due to small patient numbers in individual centres, lack of standardized charting, and difficulties accessing medical records. As electronic medical records become more widely available, this could potentially facilitate validation studies in the outpatient setting.

## Conclusions

Numerous studies about the diagnostic validity of administrative health data for the adult population have been published [7], but studies about the pediatric population have been limited in number and scope, despite the fact that diagnoses may not be equally valid in both populations. An increasing number of children are living with chronic conditions. Administrative health data can be used to estimate the burden of these conditions and provide long-term outcomes data for studies about mortality, health care utilization, and comorbid conditions. In order for administrative data to serve these purposes, their validity must be established. Our scoping review of published literature on diagnostic validity of administrative health data in the pediatric population revealed multiple gaps in the pediatric literature. Common chronic pediatric conditions have not been validated in a multiple settings, the number of validation studies decreased with increasing age within the pediatric population, and although many pediatric conditions are treated primarily in an outpatient setting, validation studies in this setting are lacking. Further studies are needed to examine validity for a broad spectrum of pediatric health conditions, in outpatient populations, and in both younger and older age groups.

## Competing interests

The authors declare that they do not have any competing interests.

## Authors’ contributions

NJS, LML, CB conceived the study and participated in its design. CB developed and conducted the literature search strategy. NJS and SJ conducted the data extraction. NJS, SJ, and LML carried out the statistical analyses. NJS, CB, and LML drafted the manuscript. All authors read and approved the final manuscript.

## Pre-publication history

The pre-publication history for this paper can be accessed here:

http://www.biomedcentral.com/1472-6963/14/236/prepub
